# Observations on Tuberculous Consumption; Containing New Views on the Nature, Pathology, and Cure of That Disease, Being an Attempt to Found Its Treatment on Rational Principles, Deduced from Physiology and Confirmed by Extensive Application

**Published:** 1842-07

**Authors:** 


### Art. XVI.

*Observations on Tuberculous Consumption; containing new views on the Nature, Pathology, and Cure of that Disease, being an attempt to found its Treatment on Rational Principles, deduced from Physiology and confirmed by extensive application. Illustrated by coloured drawings.* By J. S. Campbell, m.d., Senior Physician to the St. Marylebone General Dispensary, &c.—*London*, 1841. 8vo, pp. 404.

On our first rapid survey of the pages of Dr. Campbell's work, our eye fell upon a passage, which might very fairly have been placed in the title-page as the epigraph of the volume, so thoroughly does its spirit represent that pervading the matter with which it is associated. "He who founds his conviction in medical investigation," says Dr. Campbell, "on physical evidence alone, must be content to remain a sceptic on more questions than the present." Considered simply and abstractedly, nothing can be more true than this; no proposition could be pointed out which we should be less disposed to contest: the *animus* in which it is to be understood, however, makes all the difference in the world; and, like many other noted epigraphs, its real signification, had the author adopted it for his motto, must have been sought in the chapters that followed. Having now perused and closely examined the volume, we are enabled to interpret, as follows, the phrase we have quoted: "It is essential that an explanation be found for every phenomenon, and a theory framed to promote the understanding of every class of facts which are presumed to have been observed or to exist, (whether they actually have been so observed, or do really so exist, is a matter of very second-rate importance;) if 'physical evidence' of a direct kind be obtainable in support of such explanation or such theory, well and good, let it be given; but if not, let the investigator not be disheartened, experiments can be made, and facts can be tortured, and speculations can be dressed in plausible gear, and where is the theory which will refuse to stand when propped up, with the help of a little ingenuity, by such solid and imposing stays as these?" Such is the faith which guides Dr. Campbell through his "new views on the nature, pathology, and cure [!]* of tuberculous consumption;" a faith which in our minds, and in the minds of all those who with us believe that in pure observation and pure induction lies the true, the only means of advancing medicine,—is among the clearest and least endurable heresies. To dilate upon the comparative merits of *à priori* and *à posteriori* reasoning, however, is as much beside our present purpose, as the undertaking itself would argue instability of conviction upon, perhaps, the most perfectly established doctrine in the whole domain of philosophy; and we shall therefore, instead of indulging in platitudes on the subject, pass at once to detailed examination of the work of which we have now conscientiously, and as we believe correctly prefigured the prevailing spirit.

The author sets out with an introduction upon the "connexion between blood and life." Here we find the importance of arterialized blood for the maintenance of life dwelt upon, if not with much novelty of view, at least with much vigour of purpose. Originality may, however, be claimed within certain limits for the following notion: "One fact," says the author, "which seems to strengthen the idea that the red blood really carries from the lungs to the system, a 'something' which is the *cause* [the italics are Dr. Campbell's,] of life, is established by the ligature of a large artery. If the arterial current be arrested in its course by the application of two ligatures, the portion of blood between them speedily looses its redness, and becomes black. Are we to suppose that the life-giving ingredient has evaporated by the coats of the vessel when prevented executing the duty it is destined to serve?" The cause of life is thus made an actual and material entity; elsewhere in these pages, the inclination seems to be to regard it as a condition or effect, "evolved by the reciprocal action of the fluids and solids." With speculations of this stamp we are not disposed to delay, and refer those, for whom they may possess interest, to the original volume. We pass on to certain passages propounding doctrines, as avowedly deduced from previous statements, which are declared to have close connexion with the practical tenets of the book.

"It would appear then," says Dr. Campbell, "from all which has been said, that the varying velocities of the heart and lungs, and the varying celerity with which the functions of these organs are augmented in different individuals, are attributable to one of two causes, each dependant on the relations in which the lung stands to the system as regards either extent of surface, or capacity of action. In one case the pulse and respirations are always relatively quick, and easily accelerated, because the systemic vessels, either from inherent excitability to natural action from slight causes, or from morbid increments of action, as in fever, inflammation, &c., consume in a stated time a relatively larger quantity of red blood than the lungs, without undue action, can create, or the heart, without undue action, distribute. In the other case these functions are unusu-

* Upon Dr. Campbell's meaning of the word "cure" more hereafter.

ally readily accelerated, because, without the existence of any undue extension of systemic action, the pulmonic surfaces, whether from limitation of surface consequent on disease, or from presenting a natural structural disproportion to the body, are incapable of meeting, without inordinate increase of their functions, such quantity of red blood as the system consumes. Finally, we consider that there may exist a third cause of morbid acceleration of the functions alluded to, having its seat partly in altered organization of the lung, and partly in a changed condition of the arterial blood consequent on this, by which that fluid is so deteriorated in its vital properties, that a larger quantity is demanded to sustain a stated quantity of action, than when it is presented to the aortic capillaries in a perfect state." (p. 73.)

The author proceeds to "a short notice of *one* of the causes which appear to influence the transmission of the red blood." This cause he believes to be "a moving force resident at the circumference of the arterial tree," a doctrine which, as he observes, has been taught more or less distinctly by Bordeu, Bichat, C. Bell, and Hunter. Their doctrine of the independence of the capillary system as an agent in the accomplishment of the circulation, if not novel in itself, is however supported by novel argument. We ascertain by the microscope, the author alleges, that in the "final vessels" of the body, "the transmission of blood is accompanied by an alternate contraction and relaxation of the vessels along which it flows." In what manner of animal Dr. Campbell may have made this observation, we are puzzled to divine; we recommend to his consideration page 231 of the first volume of the translation of Müller's Physiology, wherein he will discover that the experience of persons somewhat versed in the use of the microscope, is rather at variance with his announcement. The fact is, that wherever arguments are to be found in favour of intrinsic circulatory force in the capillary tubes, they are not to be found in the *microscopical* examination of their conditions of being. "The capillary vessels," observes Müller, "present not the slightest change in their diameter, when the animal under observation is tranquil." Assuming, however, the fact as unopen to objection, Dr. Campbell proceeds, in accordance with the spirit of the epigraph we would recommend for his second edition, to lay down the cause of the said alternate contraction and relaxation. This is "the occurrence, at each motion of each minute vessel, of an entire or partial vacuum within its cylinder. And," continues this ready speculator, "when we come to consider that even a small artery divides into *many thousand branches*, it is easily conceivable how the aggregate influence exerted by those should largely contribute, on physical principles alone, to the progress of the red blood, however small the power exerted by any one may be."

The reality of this vacuum (would the reader believe it?) is forthwith assumed as established, and its cause in turn sought for. Hence the author's difficulty is one of selection; he suffers from a positive *embarras de choix;* for two efficient explanations present themselves at once: one of these, however, he inclines to adopt on the force of an analogy borrowed from the condition of the larger arteries. Those anxious to learn what the chosen explanation is, may gratify their curiosity by a reference to the 79th page of the work. We confess these *nugæ physiologicæ* (for such do the gravest questions appertaining to animal function become when handled in speculative mood alone) are to us not only without attraction, but positively repulsive. The dignity of science is offended by their promulgation as established positions, and the advancement of real knowledge retarded in the precise ratio of the hold they take of general opinion. This is said quite irrespectively of the correctness or fallacy of the inferences to which they lead, either in the present or in any other instances; these inferences, of course, may or may not be in conformity with the reality of things, just as *chance* wills they shall be.

So much for the introductory matter of Dr. Campbell's volume. In the early pages of the pathological division, we find him stating the signification in which he understands the term phthisis. He limits its application to the disease marked by the deposition of tuberculous matter in the lungs, according herein with modern usage, and at once proceeds to an examination of the three opinions which have been held respecting the origin of tubercle.

We have some doubts of the utility of resuscitating the long-buried question of the "inflammatory origin of tubercle;" but whatever be the view taken of the propriety of arguing the point, there can scarcely, we think, be any difference of opinion as to the radical imperfection of Dr. Campbell's mode of conducting the investigation. He manages to omit the only evidence affording irresistible proof that inflammation is not a necessary condition in the evolution of tubercle, namely, the clinical results obtained and promulgated by M. Louis: results made so accessible to all by the periodical journals and otherwise, that we should have fancied them familiar to very "babes and sucklings" in pathology. Here is, it must be allowed, a serious error of omission; and we are very much disposed to regard as one of commission the introduction of M. Cruveilhier's statement respecting his facts in the generation of tubercle by the injection of mercury into the bronchi of animals. What is the object of bringing once more into the light fallacies which the common sense of mankind had long consigned to obscurity? Had there been any shadow of doubt as to the fact of the imagined tubercle produced in the experiments of that pathologist being anything more than pus, had one conscientious opinion appeared in defence of the startling mistake he committed in taking common abscesses for agglomerations of tuberculous matter, then might Dr. Campbell's repetition of the oft-repeated experiment have been valuable. As it is, he has added nothing really new to what was already established. Yet there appears an affectation of importance in the announcement of these experiments by Dr. Campbell, as if the world had deemed them actually required for the settlement of debated points. And in the same spirit we find an entire page teaching, in a tone appearing to assume novelty for the idea, that judicious practice requires a strict attention to the distinction of tuberculous cases, accompanied or not with inflammatory action. Who is ignorant of this? There is not a British author who may claim any acquaintance with the subject of phthisis who has not inculcated the principle, nor, we venture to affirm, a properly-educated British practitioner who does not uniformly act upon it.

Dr. Campbell next enquires, Does tubercle originate in an error of the function of "perspiratory secretion?" That it does, is the opinion held by Andral. Our author refuses to admit this doctrine, on the ground that if so, tubercle of the skin should be extremely frequent, whereas its extraordinary rarity is matter of notoriety. The perspiratory secretion, it is true, goes forward in all the cellular tissues of the body, but it is infinitely less active in these than in the skin: if, then, we admit tubercle to be a result of an error in the function of perspiration, we have the difficulty to contend with, that the effects of the error are most frequently observed in those situations in which the healthy function is least exercised. Such is Dr. Campbell's argument. But why not suppose that the perspiratory function more readily becomes disordered in the cellular tissue than in the skin? Hypothesis is the order of the day, and this, which seems quite as good as its fellows, gets over the difficulty in a very satisfactory manner.

The third opinion as to the origin of tubercle, an opinion which has been generally adopted, and which received the assent of Laennec, is that it is the result of some error in the process of nutrition. Dr. Campbell assumes the advocacy of this doctrine, but is not content with the simple statement of arguments calculated to support it; he justly observes that Laennec, no less than all others, has left us entirely uninformed, not only as to how this error is remotely produced, but also as to the immediate mode in which it determines the deposit of the foreign matter. Of these points he proposes to attempt the elucidation. In this attempt lie the novelty and the main claim to attention of Dr. Campbell's book, and we shall here, therefore, accompany the author closely.

In accordance with the arrangement adopted by Dr. Campbell, the first point for enquiry is the "nature of that action originating apparently in the system, and directed towards the lung, of which the deposit of tubercles in the latter organ is the result." Two important points are necessarily involved in such an enquiry. First, "the nature and character of the primary constitutional disturbance which tends to the production of the local affection; and secondly, the mode in which this actually leads to its establishment." The first involves the consideration of the phthisical diathesis, the tuberculous cachexia of Sir James Clark; the second may with equal propriety, observes the author, embrace a consideration of the physical characters of tubercles, the probable mode of their formation, and the tissue which they occupy. Dr. Campbell begins with the last-mentioned subject, by an examination of the question of the identity of pulmonary granulations and crude yellow tubercle. Having stated the arguments adduced by Laennec, and some of those of Louis, in favour of that identity, the author enumerates the counter-arguments of Chomel and Andral. Appearing to consider these latter satisfactory, he yet "desires to add another, derived from the action of reagents in the first place, and the simple process of decomposition in the second, on lungs which present the two varieties of morbid growth." When slices of lung are placed in water for a length of time, he observes, and the bottle is frequently agitated, they ultimately form by the process of natural decomposition a dark slimy solution, with fibres or threads diffused through it—the coats of vessels or minute bronchial tubes. Now when the lung contains semi-transparent granules, these are found to decompose with as much facility as the natural tissue, while crude tubercles may by repeated maceration be separated from the pulmonary substance in the perfect state, as regards colour, shape, and consistence. This difference between the gray granulation and crude tubercle in respect of proneness to putrefaction appears to Dr. Campbell to strengthen the arguments in favour of their non-identity, but he admits that it by no means proves it. It might be argued that the component material was in both cases the same, but in its early stage the granulation too soft to resist effectually an amount of maceration exercising no notable influence on it when fully consolidated. The action of alkalies points, likewise, to some difference in the pulmonary granulation and the crude tubercle: the former is simply softened and swelled, without any other change in its original appearance, while yellow tubercle forms a clear saponaceous solution. To our minds these facts, for such we presume they are, prove nothing more as to the fundamental question than does simple inspection of the two substances. That there is a difference apparent on the simplest examination is as obvious as the day, and it would be infinitely more extraordinary if the agents mentioned by Dr. Campbell acted in the same way on two substances of such strikingly different aspects. But this difference of aspect furnishes no shadow of proof that one is not an advanced stage of the other, and the chemical difference by the author's experiments fails as completely to offer such demonstration. The chemical composition of cartilage and bone differs, yet it will not be denied that the former constitutes the first stage of the latter; or, in other words, the necessary stroma for its development. This is all which is contended for by Laennec and M. Louis in respect of the relationship of the gray granulation and yellow tubercle; and Dr. Campbell's experiments modify in nowise, that we can perceive, the terms of the question. He starts, however, with the notion of his having proved satisfactorily that the gray semi-transparent granulation has no necessary connexion with pulmonary tubercle, but that this latter substance *first* shows itself in the lung as a yellowish friable matter, &c. To its precise characters we shall by and bye have to return. Meanwhile we pass with Dr. Campbell to what he rather obscurely and affectedly terms "the question of tubercular locality;" that is, the determination of the immediate seat of the deposition of tuberculous matter.

To the probability of the opinion of Dr. Carswell that tubercle occupies the air-cells, he raises the "serious objection" stated by Lombard, namely, that as those cavities communicate with the trachea, their contents would necessarily be occasionally detached and "extruded" in the early stage, "a thing well known never to take place until they have passed into a softened state." Dr. Campbell considers this objection strengthened by the fact, that the matter of tubercle adheres with much tenacity to the lung when first formed, a point in their anatomy easily ascertainable by the examination of a tuberculous organ. Notwithstanding these objections, our author still inclines to the conclusion that tubercle is "usually, if not always, associated with the true breathing portion of the lung:" the motives for this rather paradoxical inclination, as it is at least under the circumstances, are stated to be "the form of tubercles, and many other circumstances connected with their history." Then arises, it is admitted, the question, whether any means may be found "of reconciling this opinion with the obvious difficulties to which it is liable."

Andral many years since pointed out a fact in the anatomy of phthisis which may be readily verified. If a tuberculous lung be insufflated, dried, and sliced into layers, certain air-cells with dilated cavities may be seen, having thickened walls, which present a peculiar yellow tinge; this tinge is deeper in some points than others, and by care a "number of minute yellow round bodies, which are evidently tubercles," may be distinguished in the thickened parietes. The yellow tinge Andral looks upon as a change "preceding the secretion of tubercles;" Dr. Campbell regards it as produced by bona-fide tuberculous matter. This matter he believes to be seated in the interior of the vessels ramifying in the walls of the vesicles; he could often perceive by the aid of a moderately strong glass, lines of yellowish vessels pervading the lining membrane of the air-cells. The yellow matter is with difficulty detached from the membrane; and Dr. Campbell's "conviction" is, that this depends on its being actually contained within the vessels.

Here is the prominent idea in this author's volume: he believes that tuberculous matter, instead of being effused from the blood-vessels into the inter-vascular spaces at its origin, is retained within them, and that, by the accumulation of this matter in several adjoining vessels, a mass is at length produced sufficiently large to attract the naked eye,—in other words, "a tubercle" is formed. We shall state, as plainly as we can, the substance of the evidence brought forward in support of this doctrine. The discovery, as alleged, of "yellowish vessels" has, we confess, little importance in our eyes: Dr. Campbell saw yellow *streaks*, and calls them *vessels*,—and this is all.

"When a minute tubercle is submitted to the microscope, it is readily perceived not to be the homogeneous nodule which it appears to the naked eye; it presents a round surface, made up of other round particles or molecules, which adhere together by a delicate filamentous membrane; occasionally these molecules appear to make lines, forming a chain of soft hair-like threads, with round swellings at certain distances, so interlaced with other lines of the same description as to render their isolation under the eye very imperfect, but still distinct enough to convey the idea of a body formed of innumerable molecules adhering to each other, not as a consequence of their own cohesion only, but through the intervention of a tissue which forms their seat." (p. 140.)

These observations are stated to be confirmatory of others made by Kuhn, the allusion to whose inquiries is followed up by the *naïf* remark, that "the idea does not seem to have occurred to this observer that the globules he describes might possibly be extremely minute particles of a white matter, contained within the caliber of small vessels, whose coats formed the delicate line connecting them. And yet, if he had endeavoured, from imagination only, to describe the probable appearance of the inner membrane of an air-vesicle, whose minute vessels were obstructed by an abnormal deposit, he could not have conveyed a clearer picture." Dr. Campbell, himself, so clearly states the inadequacy of any observations of the stamp of those just detailed, that he will not assert their power of proving his position; yet affirms that, coupled with the arguments before brought forward, they go far to support the probability of the opinion. Under these circumstances, it is unnecessary for us to show, what indeed must be obvious to the reader, that they are incapable alone of demonstrating the point at issue; of the "arguments before brought forward," we have already disposed.

It is known, observes Dr. Campbell, that a certain relation exists in the healthy state between the caliber of the capillary vessels and the bulk of the blood-particles ; that if substances capable of thickening the blood be injected into the veins, death ensues, and is very probably caused by an obstruction arising in the extreme vessels of the pulmonic circle. Here the obstructing cause is probably general; were it partial, it is inferred that a sufficient number of vessels would remain pervious to maintain the purposes of respiration. But the normal relation above referred to may, *conceivably*, be altered in two ways: that is, the vessels may be too small, or the particles may be too large. With these facts and conceptions to work upon, Dr. Campbell has instituted a series of experiments on injection of the lungs after death ; " and if it can be shown by these that there exists a vast difference in the facility of transit of the same compound injection, though the capillaries of different lungs,—that this facility is least when the evidences of tubercle are most distinct, and much diminished where the evidence of *tendency* to tubercle, as derived from other sources, is considerable; then do I conceive that facts have been adduced corroborative, to a great extent, of the opinion, on other grounds maintained, that tubercle is the result of obstruction in the minute vessels of the pulmonary circulation." The injection employed was composed of mutton suet, with a fifth of olive oil, and some vermilion, and was found to pass with facility through the pulmonary artery, and through the veins into the left auricle, in some instances having undergone no change in the transit, in others having lost some of its colouring matter. The loss of colouring matter being only apparent in the injected material in the auricle and veins, Dr. Campbell ascribes its absence there to retention by the capillary vessels, whenever their caliber falls below the ordinary standard, either in the entire pulmonary system or in limited parts of this. There is one rather important consideration which Dr. Campbell seems to have very fortunately, at least for the existence of his theory, forgotten ; and this is the simple fact, well known to practical anatomists, that nothing is more common than to observe the vessels of an injected limb deficiently supplied with or altogether free from, in certain points of their course, the colouring particles which have been used for the manufacture of the injected material. To infer that this sort of filtration depends upon a narrowness of capillaries, itself evidence of tuberculous constitution, would be at variance altogether with observed facts ; the phenomena referred to having been witnessed in subjects of every variety of temperament, and enjoying every variety of health.

These matters, however, being established to the author's satisfaction, a new point for investigation presents itself; namely, " whether the relative facility with which the constituents of an injection are separated in different cases, is susceptible of being connected with appreciable peculiarities of constitution, in the individuals whose lungs form the subjects of examination." Dr. Campbell seems aware of the large number of observations which would be required for the settlement of the question here started, and he admits that what he has to say on the subject is more to be considered as proposing than determining it. He has subjected the lungs of thirty-eight individuals to the process of injection, some of them healthy, the greater number variously diseased. In examples where no perceptible disease of the lung existed, and where it is alleged " the individual showed no signs of a strumous constitution," the red injection entered by the pulmonary artery and returned through the corresponding veins, having minutely filled the extreme vessels. In a subject of strumous constitution, whose lungs were nevertheless, "as far as could be traced," free from tubercle, (a child aged four years,) the majority of the pulmonary veins were filled with the red matter, altogether unaltered, while in some instances they contained the white portion only. In a child aged five, who died of tuberculous softening of the brain, and whose lungs were besides studded with tubercle, the quantity of injection deprived of its colouring material was considerably greater. From these facts, as well as others which he does not think it necessary to refer to, Dr. Campbell concludes that "throughout the series a marked difference has appeared in the extent of separative influence exercised by the pulmonary vessels of different subjects; and that this has borne a strict relation either to the existing tubercular disease of the organ, or to the constitutional tendency to phthisis deducible from evidence less conclusive."

The variable degree of facility with which particles of a certain size traverse the capillary vessels of the lung being admitted, it remains to be enquired how this applies to the formation of tubercles, or throws any light on the nature of that condition which has been conceived to establish a tendency or predisposition to their evolution. This is simple: grant that the pulmonary capillaries of strumous subjects are of unusually small caliber, generally or partially; and admit that particles foreign to the healthy nature of the blood occasionally exist in that fluid, and nothing can be more clear than that the smallness of the capillary bore must constitute the so-called "predisposition" to pulmonary *tuberculization*, while the actual formation of *tubercles* will depend, even in a subject having blood impregnated with tuberculous matter, more immediately upon the degree of smallness of the capillary vessels, than upon the amount of that tuberculous impregnation.

Two objections, it is remarked in anticipation by Dr. Campbell, may be urged to this mechanical theory of the formation of tubercles out of tuberculous matter. The first is, that "the theory leaves it unexplained how such an abnormal condition of the pulmonic capillaries should occur at all; and still less how it should only be found in some portions of those which pervade the same organ." This objection we think of comparatively no importance; the great point is, whether the alleged abnormal condition exists at all; if it do, we need care little for the actual cause. But the second is an objection of more weight: "the very existence of the globules which we have supposed to be the determining cause of the disease is, so far as we have yet gone, assumed, but not proved." Dr. Campbell seeks to divest this objection of its force by allusions to the growing belief in the soundness of humoral pathology; to the notion vaguely expressed by Andral, that tubercle may be liquid at the moment of deposition, and rather more definitely affirmed by Cruveilhier; and to the recognition by Dr. Carswell of the blood as one of the localities in which tubercular matter is occasionally formed. We confess ourselves unable to detect any semblance of proof in these surmises of various writers; and how the fact that humoralism is gradually ousting its rival solidism from the field of pathology, advances *demonstratively* the views which Dr. Campbell undertakes to establish, we are, sincerely speaking, unable to perceive. Still we have not exhausted the author's budget of reasons for teaching the origin of tubercle to be in the blood : "the strongest facts in its favour are undoubtedly those furnished by the microscope." The facts here referred to are, that, "under some conditions of disease actually formed, or in some states approaching to disease," particles are found in the blood different from those which belong to it in health. These have been called pus-globules, but their name Dr. Campbell thinks a bad one, because it identifies, or rather attempts to identify, the nature of a fluid of which the character is unknown. The important point in connexion with them he believes to be, that they have hitherto been found chiefly, if not solely, in the blood of persons who labour under some form of cachexia, and hence frequently when no decided symptoms of phthisis have been present; "but, on the other hand, where the tendency of this disease is strong or its presence unequivocally announced by decided signs, they will, *I am disposed to think,* be seldom absent; and they thus appear to constitute one of the elements demanded for its full and perfect formation." We cannot take the favorable view of the importance of this train of argument evidently taken by its author: beyond the announcement of a *remote possibility,* we can detect nothing in this exposition of his opinions.

Dr. Campbell continues his investigation, however, as if his path were smooth before him : having passed from the formation of tubercles to the accumulation of tuberculous matter in the capillaries, and from this to its primary and invariable existence in the blood, his ambition is to rise a step further, and determine the cause of such existence. This he finds in a "primary error of digestion which lies at a point in its series of actions anterior to that of final sanguification"—by the term digestion being understood the sum of changes undergone by the food from its first reception into the stomach to its final identification with blood by the respiratory function. This connexion of indigestion with the development of tubercles has, as is well known, been taught by others; Dr. Campbell gives no new evidence of the correctness of the doctrine, but, in commenting upon it, makes a statement respecting the history of phthisis, practically considered, which we cannot suffer to pass unnoticed. He speaks as of an every-day occurrence of meeting persons "exhibiting, perhaps, all the characters of the diathesis, but in whom the symptoms are still insufficient to justify more than a strong suspicion [of phthisis;] and the physical signs are *still less decided.*" Now, we affirm, on the contrary, that cases where the physical signs are less decided of the existence of tubercles than the symptoms, are of most extraordinary rarity; nor can we conceive the assertion of Dr. Campbell to be the deliberate convictions of any man who has carefully availed himself, in every case submitted to his examination, of the numerous sources of information supplied by physical investigation in its existing state of advanced culture. But, as we shall hereafter find, Dr. Campbell's *forte* does not lie in his familiarity with the niceties of physical diagnosis.

But for the theory: let us consider its successive steps, and note, as we pass, the degrees of solidity of each. First, tuberculous matter stagnates in the capillary vessels of the pulmonary air-cells; of this, as we have before said, we can discover no proof but that Dr. Campbell and others have seen yellowish matter or specks in the walls of the aircells, and that Dr. Campbell considers it advisable to call these specks vessels. Secondly, tubercles appear to be formed of a number of these vessels containing tuberculous matter in the mode just adverted to; it is sufficient to say that the appearances, here referred to vessels crammed with tuberculous matter, have been by others—or at least by one other observer—described, on quite as good grounds, to be produced by the interlacement of the filaments of a species of conferva! Thirdly, the tuberculous matter stagnates in the pulmonary capillaries, because, in scrofulous subjects, they are of morbidly small caliber; the evidence of this being the result of certain injections with suet and vermilion performed on the lungs of healthy and diseased persons. The answer to this view we have already given. Fourthly, tuberculous matter exists from the first in the blood, and its presence there is a necessary condition of the formation of tubercles. The only proof attempted to be adduced of this most important notion is, that "pus-globules" have been found by certain manipulators with the microscope in the blood of cachectic subjects; and the proof amounts simply to this, that Dr. Campbell finds it convenient to consider certain particles—of which he himself affirms no one knows the real nature—the primary constituent elements of tubercles.

If this be not a fair exposition of the leading points of Dr. Campbell's theory, we regret it, and can at all events declare that our wish has been to represent his hypotheses as they appear under his sanction. If, on the contrary—as we believe is the fact—we have correctly described them, then can we only pleasurably anticipate the satisfaction of the solid pathologist, who may for the first time have met with them in these pages, as he discovers this new evidence, that flimsy though ingenious speculation cannot stand in lieu of sound observation and cautious induction.

In the chapter describing "the causes and character of the morbid reactions exerted by tuberculous lungs on the organs and functions generally," the same tendency to speculation encounters us in almost every page; this is the more to be regretted as there is here much matter of a practical kind worthy of attentive study. We cannot afford space for the consideration of the topics therein touched upon, and pass to the doctrines upon the treatment of the malady in its "formative stage," at the commencement of which we find the author discoursing upon the signification of the term *cure*.

"In its broadest sense, as usually employed, the word *cure* appears to express the *complete* restoration of a part or of the whole body, which had previously erred, either in function or structure, to a normal state; and this without its necessarily implying any connexion between appreciable cause and effect, inasmuch as such restoration is frequently induced without our being capable of explaining it. But again, the term is commonly and, as regards the medical art, more properly used to express a direct connexion between the means producing and the effect produced—to indicate not only the fact of restoration, but the agencies, be they medicinal or dietetic, on which the change is presumed to depend; and it is in this sense that I mean to employ it in the succeeding pages............... But whilst we thus define the term cure as in strictness implying the *entire* restoration of function and organization, there exists a more modified sense in which it may be and often is employed. Perfect integrity of organs or perfect integrity of action in these is found, it may safely be averred, in very few human beings after life has a little advanced, and it hence follows, that the term may be legitimately used to express, not indeed complete restoration, but only such an approach to this as enables the individual, under certain limitations and modes of management, to carry on life without inconvenience even to a protracted period." (pp. 221-2.)

We deny that the term *can* be legitimately used in any such sense, and we resist its employment thus on logical, common-sense, and moral grounds. We resist it on logical grounds, because it is a palpaple absurdity to define by the same word two things which, as is distinctly admitted and as could not be glozed over, are essentially different. We resist it on common-sense grounds, because common sense and the experience of mankind concur in showing that, even understood in the latter of the two senses stated, a cure of phthisis (though for inexplicable reasons an occasional *chance* result,) cannot be regarded in the remotest degree as a consequence, predicable *à priori*, of any mode of treatment, however judicious and however sustained. And finally, we resist it upon moral grounds, because the blazonment of the phrase "cure of consumption" in the title-page of a book and the columns of a newspaper is—viewing the matter in the most favorable aspect possible for those who authorize both—a heartless cajolery of the myriad of sufferers from the disease; a mockery of those who, interpreting the word as their dictionary and common usage teach, straightway imagine that at length a panacea has been found for the malady that destroys them, and eventually sink more rapidly to the grave from the sudden prostration of the hopes they were deceived into cherishing.

The indications of a "cure" derivable from the views of Dr. Campbell respecting the malady are, he states, these:

"First, to counteract that morbid state of the digestive organs, without which tubercles cannot be produced. Secondly, to accomplish a solution of this matter after it has passed into the blood, and thus arrest its local deposition, presumed to depend on mechanical retention in the extreme vessels of the pulmonary artery. Thirdly, to place and retain the patient under such circumstances, in reference to his medical, dietetic, and general treatment, as seem on rational principles best calculated to meet those evils which result from the existing state of his respiratory organs; thus affording time for removal by the natural process of softening of such tubercles as already actually exist." (p. 225.)

These indications are clearly stated: in the second only of them is there any character of novelty, and to the details referring to it our observations shall be almost altogether confined. First, however, we must take the trouble of pointing out an error of no mean importance connected with the subject of diathesis—an error which, like most of those committed by Dr. Campbell, originates in his unfortunate proneness to submit the investigation of matters of fact to speculation, and not to observation. The phthisical diathesis is the subject; by which Dr. Campbell means the constitutional state of subjects destined to become consumptive; and *more solito* the outward aspect of these individuals is portrayed. Here we have the delicate and clear skin, the light hair, the elegant contour of the face, body, and limbs, the tumid upper lip, the large and lustrous eyes, the pearly sclerotic, with the sound and regular teeth, the "blush suffusing the cheek of beauty," &c., which it has been the fashion to describe in more or less glowing periods as the outward and visible signs of tuberculous constitutions. But though these pictures may be pretty, it does not exactly follow that they are correct; our own experience (and we could give this numerically,) cries loudly against their accuracy; but we prefer referring to results already before the public. We read in the elaborate and, in many respects, admirable work of M. Fournet: "I have seen *very few* phthisical patients with light hair; in *almost all* of them the pilous system was well developed and brown. I have met with several whose hair was ebony black. The colour of the iris agreed very accurately with that of the hair. Hazel or brown eyes were most frequent among my patients." Simply, no doubt, because the hazel is the most common French eye. Again, "the occurrence in phthisis in subjects endowed with a strong, muscular constitution, having a well-developed and well-formed skeleton and sanguineous temperament is more frequent, on the evidence of my cases, than is commonly supposed. This class of subjects forms nearly *one third* of the total number of phthisical patients." Now is it probable—is it even remotely probable—that, taking the entire mass of individuals entering the Parisian hospitals, and afflicted with all varieties of disease, we should find more than one third possessed of muscular forms and the sanguineous temperament? On the contrary, it is certainly improbable, to say the least, that that third would be found; but we shall not on this account infer that the temperament in question forms *the* or even *a* predisposition to phthisis. No; all that the facts authorize us in affirming is, that subjects of every constitution in existence are liable to the inroads of phthisis to the same or nearly the same amount. The error which Dr. Campbell has thought fit to perpetuate in emphatic terms is one not merely important as a point of pathological doctrine, but one likely to entail practical mischief; had this been otherwise, we should not have considered it deserving of so lengthened a notice.

Dr. Campbell's second indication, we have seen, is to "accomplish a solution of tuberculous matter after it has passed into the blood;" no trifling undertaking, it would appear, on first thought. Our author was not, however, discouraged by the apparent difficulty of the task. He examined experimentally the degrees of "solvent power exerted towards the matter of tubercle by various substances." The pure alkalis were in these experiments, as has appeared in another part of this notice, discovered to be the most effective agents of solution. Many other circumstances seemed to Dr. Campbell to point out these chemical agents as deserving of a more persevering trial in the treatment of phthisis than they had yet obtained. Among these circumstances the author mentions their "universally-acknowledged efficacy in the analogous affection of external scrofula, and the general impression which prevailed in the humoral schools of pathology, that they constituted the most effectual of those substances which were comprised in the class of attenuating medicines." The alkalis have besides, as Dr. Campbell correctly states, been mentioned by almost every writer on the medicinal treatment of consumption as worthy of more or less confidence. Now, if we consider the grounds upon which the use of alkalis is here advocated, we shall not find, we apprehend, very strong motives for estimating the practice highly. The very notion that tuberculous matter exists in the blood—the fundamental notion or basis on which the superstructure is raised—is, at the best, a possibility, in reality a mere hypothesis. But, admitting that it does so exist, and admitting the equally-necessary postulate, that tuberculous matter is readily dissolved out of the body by the fixed alkalis, does it follow, that when within the body and contained in the blood-vessels, the ingestion of alkalis into the stomach will similarly affect it? Not in the very remotest degree. But this is not all. Let us again admit that it will actually so disintegrate and dissolve tuberculous matter accumulated into small masses in the lungs: have we any reason to anticipate benefit from such disintegration and solution? What we should really do in this case would be to spread through the whole system in a semi-liquid state, and by means of the blood, what was previously aggregated in one or more limited spots. Would the diffusion of pus or of the matter of cancer through the frame of persons suffering from abscess or carcinoma—granting we had the power to effect it—be likely to improve their condition? These points appear to us to prove most clearly that, considered upon *à priori* grounds, the alkaline treatment possesses no shadow of a claim to the attention of solid men.

But Dr. Campbell's advocacy assumes a very different tone of importance when he goes on to inform us that a "very ample experience, extending through a period of nearly ten years, and embracing above 400 cases of well-marked phthisis, confirmed the soundness of the views which originally led to its adoption." Yet even here there is a palpable *non sequitur*. It does not in truth follow, supposing we admit that these 400 cases were all of them really cases of well-marked phthisis, that they were all treated with alkalis, and that every single consumptive individual was distinctly and materially benefited by their use—it does not follow, we say, even in the most distant degree, that Dr. Campbell's views, or any other views which might have led to the adoption of the alkaline treatment, were sound: it simply and alone follows, that phthisical subjects derive benefit from the use of alkaline medicines. And here is, in truth, the important and really interesting question; is improvement actually obtainable and capable of being sustained under the constitutional influence of these medicines? The reader will perceive that we speak not of "cure;" we put the question in the most favorable manner possible for Dr. Campbell; we ask simply, has *sure*, or even tolerably sure, and *continued* amelioration of consumptive subjects arisen under this treatment? not such amelioration as will, we all know, frequently follow, *pro tempore*, in the case of dispensary patients, an altered system of diet and the use of ipecacuana or any other simple medicine. Dr. C. refers us to some cases at the end of his volume in proof of even much more—of complete suspension, at the least, of the disease having followed the exhibition of alkalis; and to these cases we accordingly turn.

Dr. Campbell observes that, to render reported cases of any value, two things are required: honesty on the part of the reporter, and sufficient evidence that he has not been himself deceived. Of the first of these Dr. Campbell is, we are persuaded, possessed. As respects the latter, it is not sufficient that the *terms* of evidence should exist in the report of any case, the reader also requires (unless the fulness and manner of the description be such as to make this unnecessary, which is the rarest of merits in the works of authors) to know from other sources whether the writer be really capable of accurately appreciating the conditions which supply those terms. On carefully considering Dr. Campbell's efficiency in this point of view, on the grounds furnished for the formation of an opinion in some introductory remarks on diagnosis in the chapter containing his reported cases, our inference is that his acquaintance with the physical signs of the disease is correct generally, so far as it goes, but far from minute;* but it will be perceived that, in one point of view, the want of either the power or the will to discriminate very minute changes in the physical state of the lungs would militate *against* Dr. Campbell's establishment of his doctrine; it would prevent him from detecting the very earliest deposition of foreign matter in the lung, the condition most likely to be benefited by the treatment lauded. That he has scarcely attempted such detection, is manifest from the simple fact of his considering percussion a more valuable instrument in the early discovery of tubercle than auscultation.

The cases related, sixteen in number, are arranged under these heads: under the first appear "ten examples of phthisis in its early stage, which appeared to be made out in a manner tolerably satisfactory." Hence the treatment is alleged to have "conduced to the quiescence of existing pulmonary tubercles, and arrested their tendency to increase." Our space will not admit of more than a very brief commentary upon some of these cases. *No.* 1. Here the existence of tubercles is not, by any means, positively established; the patient took the alkali for two months, and passed subsequently two months without any cough, to which she had previously been subject. Dr. Campbell saw her eighteen months after she had been treated, but, strange to say, neglected to examine the chest, or at least says nothing of his having done so. *Case* 2. Alkali continued for fifteen months; signs of consolidation in both lungs, which signs had diminished on one side, not on the other, at the end of the treatment. Seven months later, Dr. Campbell saw this woman, but tells us nothing of the physical state of her chest. *Case* 3. The medicine used for two months; signs of consolidation on both sides, unchanged at the end of that period, except that the "bronchial respiration had ceased to be harsh." The meaning of this does not appear very clear. Dr. Campbell saw this woman upwards of a year afterwards, but, as usual, leaves us in ignorance of the physical state of her chest. *Case* 4. "The upper lobes of both lungs, especially the right, were extensively solidified." In two months, this man "was dismissed *well*"! He had abdominal symptoms when first seen; upon the removal of these, we presume Dr. Campbell founds the use of the adjective *well.* That the case proves, as it is alleged, the effect of the alkali in preventing a threatened fresh deposition of tubercles, would probably never have struck any one but the author himself, at least upon the evidence he has given to the public. *Case* 5. This man appears to have been temporarily better under the alkali upon two occasions, when an aggravation of his ordinary symptoms occurred. *Case* 6. Alleged signs of solidification at both summits; in six weeks the patient was "very nearly free from cough and much increased in flesh." Alkalis with anodynes. *Case* 7. Alkali for twelve months; at the end of that time no change in the physical signs. Four years after, the state of chest not enquired into, though the patient was then seen. The remaining three cases are more or less closely similar to these: in all benefit, in respect of the local and general symptoms, appears to have been effected. But we see no particle of evidence to show that such benefit depended on anything but the disappearance of local

* Dr. Campbell appears to be wholly ignorant of the diagnostic value of the expiratory murmur.

congestion; nor do we feel disposed to admit that any practitioner, who has treated phthisis extensively, could not produce many cases of quite as satisfactory character—cases which had been submitted to all varieties of treatment. It will be observed that, so far as they are mentioned, the physical signs appear to have undergone none of that modification which, were Dr. Campbell's theory of solution correct, should have been manifested along with the amelioration in respect of symptoms.

Next follow three cases, "tending to show the possibility of tubercular absorption." These cases are worth study of an attentive kind; we, therefore, refer the reader to the original reports. Lastly, appear three "cases of phthisis, in its very advanced stage, benefited by the alkali."

On the whole, we are of opinion that sufficient evidence has been here brought forward to show that alleviation of symptoms, to a marked amount, will sometimes take place under the use of alkalis, and that these medicines are consequently deserving of the attention of persons engaged much in the treatment of consumptive subjects; but we, as before intimated, can discover no distinct proof of their *extraordinary superiority* as remedial agents in the management of this malady.

The three points upon which Dr. Campbell insists, regarding the mode of administration of the alkali, are, that it be administered in the *pure state* (the liquor potassæ of the Pharmacopœia); that no medicine, which might convert it into a salt, be prescribed simultaneously; and that its employment be persevered in for a long time. The dose of the medicine, as employed by Dr. Campbell, is from ʒjs. to ʒj. three or four times daily; and the ordinary vehicle is milk or water. If acidity is present, gr. x-xx. of the carbonate are added.

In various parts of the work, with which we now part, close practical familiarity with the management of consumptive disease is distinctly exhibited; and, estimating him by this volume, through which alone we know him, we should be inclined to regard very highly Dr. Campbell's ability as a therapeutical physician.

The literary execution of the work is extremely careless. Dr. Campbell's versions of authors' names are remarkably curious: we have *Loui* for Louis; *Cattereau* for Cottereau; *Crouvillier* for Cruveilhier; *Therard* for Thénard; *Tod* for Todd. The names of medicines are scarcely less scurvily treated: hydrargerum and hyociamus startle our orthographic sense on more than one occasion. Besides these appear such words (many of them *passim*) as d*i*spnæa, d*i*spepsia, anal*a*gous, cahexia, con*j*estion, hæmopt*o*sis, *laying* in the sense of lying, shre*a*ds mucilag*e*nous, putr*i*faction, sept*œ*, &c. Nor can Dr. Campbell be considered always fortunate in the notions he appears to entertain of the meaning of words, or particularly elegant in their selection. In the preface, he speaks of the "*innate* conviction" he entertains of the importance of his volume; elsewhere, talks of a "*drummy* abdomen;" "contents himself with *preceding* the detail of cases with a brief announcement," &c. At page 5, he laments that "we are frequently compelled to *minister to diseases* in an empirical manner." Shakspeare, who probably suggested the phrase here used, is not responsible for its erroneous meaning, as it stands in Dr. Campbell's pages: Macbeth asks his doctor if he can "*minister to a mind* diseased?"—not if he can minister to a disease of the mind,—which latter query would simply mean, Canst thou add fuel to flame, and make the malady worse than it is?

